# Mobile Mapping of Sporting Event Spectators Using Bluetooth Sensors: Tour of Flanders 2011

**DOI:** 10.3390/s121014196

**Published:** 2012-10-22

**Authors:** Mathias Versichele, Tijs Neutens, Stephanie Goudeseune, Frederik van Bossche, Nico van de Weghe

**Affiliations:** Department of Geography, Ghent University, Krijgslaan 281, S8, B-9000 Ghent, Belgium; E-Mails: tijs.neutens@ugent.be (T.N.); stephanie.goudeseune@ugent.be (S.G.); frederik.vanbossche@ugent.be (F.B.); nico.vandeweghe@ugent.be (N.W.)

**Keywords:** Bluetooth tracking, mobile sensors, mobile mapping, crowd counting, crowd mapping

## Abstract

Accurate spatiotemporal information on crowds is a necessity for a better management in general and for the mitigation of potential security risks. The large numbers of individuals involved and their mobility, however, make generation of this information non-trivial. This paper proposes a novel methodology to estimate and map crowd sizes using mobile Bluetooth sensors and examines to what extent this methodology represents a valuable alternative to existing traditional crowd density estimation methods. The proposed methodology is applied in a unique case study that uses Bluetooth technology for the mobile mapping of spectators of the Tour of Flanders 2011 road cycling race. The locations of nearly 16,000 cell phones of spectators along the race course were registered and detailed views of the spatiotemporal distribution of the crowd were generated. Comparison with visual head counts from camera footage delivered a detection ratio of 13.0 ± 2.3%, making it possible to estimate the crowd size. To our knowledge, this is the first study that uses mobile Bluetooth sensors to count and map a crowd over space and time.

## 1. Introduction

Throughout human history, people have had the tendency to sometimes gather in large numbers—either in an organized or spontaneous way. The character of these gatherings or the motivations of the people present may vary from purely recreational (festivals, parades, sports), religious (pilgrimages) or political (demonstrations, inaugurations) [1]. Regardless of the expected level of agitation, large crowds will always constitute a potential security risk. Indeed, they will sometimes reach or even surpass the short-term carrying capacity of the local environment or certain bottlenecks inside it. Often caused by lacking planning and management, critical crowd densities can give rise to significant human casualties [2]. Besides these security issues, large events additionally provide significant economical opportunities [3,4]. Certain mega events such as the Olympics also have important social impacts for the hosts [5].

Whether for the purpose of crowd management or economic incentives, one of the most important (and certainly the most tangible) indicators of a crowd is its size. When access to an event is restricted (e.g., through tickets or turnstiles at access points), *counting* a crowd is trivial. For open-access events, however, this is much more challenging. Additionally, crowd size estimations often differ from reality due to subjectivity or contradicting motives of the different stakeholders [6]. Given that the success of an organized event (e.g., a protest march) is often measured by its attendance, organizers may for example be tempted to exaggerate attendance figures in order to put more weight on public opinion. Perhaps one of the most telling examples is that of the Million Man March held in 1995 in Washington DC, where depending on the source, crowd size estimations varied between 400,000 and 1.5–2 million.

Public safety cannot afford such margins of error and requires objective and accurate crowd density figures. While various methodologies (an overview is given in Section 2) have been suggested to estimate the size of a crowd in an objective manner, they often entail high levels of uncertainty and are impractical when applied in scenarios with high levels of movement. The *mapping* of a crowd, for which information is needed on the specific location or sequence of locations of individuals within the crowd, is even more complex than just *counting* and requires more advanced methodologies.

In this paper, we will present an alternative methodology for counting and mapping a crowd based on the Bluetooth technology. The usefulness of our approach will be illustrated in a case study where spectators of a road cycling race are mapped using Bluetooth sensors installed on a mobile platform moving along the track, delivering detailed spatiotemporal information on the crowd assembled for the sporting event. In Section 2, we discuss the current methodologies used to count and/or map crowds, their most important deficiencies and how Bluetooth technology might offer a valuable alternative—especially when individual mobility needs to be accounted for. Subsequently, we present a Bluetooth tracking methodology and its specific deployment in this paper in Section 3. In Section 4, we then give background information on the case study (Tour of Flanders 2011). The results of an experiment carried out prior to the cycling race, and the main case study experiment itself are outlined in Section 5. Finally, we interpret and discuss these results (Section 6) and give a short conclusion (Section 7).

## 2. Counting and Mapping a Crowd

Different methodologies have been proposed to estimate crowd sizes. First, rough estimations can be made by speculating on the basis of prior experience and knowledge of the local terrain, or manually counting either static or mobile attendees at one or more fixed locations [7]. Alternatively, secondary data sources such as the amount of waste generated by a public and public transport usage to reach an event [8] have also been used in the absence of readily available primary data. A third and more sophisticated methodology—introduced in the 60s [9] and later modified in the 70s [10]—is to carefully analyze aerial photographs of a crowd and to outline zones of uniform crowd density. Using standard density rules that are still used today (loose crowd: 1 person/m^2^, solid crowd: 2 persons/m^2^, very dense crowd: 4 persons/m^2^) and the surface areas of the outlined zones, one can estimate the total number of attendees. For the previously mentioned Million Man March (http://www.bu.edu/remotesensing/research/completed/million-man-march/), this *grid/density* methodology yielded an estimate of 870,000 people with a margin of error of about 25%. Several other studies have finally calculated crowd densities with the help of computer vision techniques on very high resolution satellite images [11] or ground-based cameras [12,13]. Despite some promising results, these techniques remain confined to laboratory conditions [14]. Hence, there is a need for a more robust methodology.

Counting a crowd gets even more challenging, when the dynamics of the crowd are to be accounted for. In the relevant literature, mobility is usually attributed to the crowd itself (e.g., a march), giving rise to a distinction between static and mobile crowds, with different counting methodologies for each of these categories [6]. Mobility can, however, also be part of a scenario with a (largely) static crowd when there is a mobile “attractor” at play (e.g., a parade or a cycling race where spectators are lined up along a linear trajectory). As such, both the mobility of the crowd and the attractor (if present) should be taken into account. Table 1 summarizes how different crowd scenarios may be formed based on the above distinctions.

The added difficulty in estimating the size of a dynamic crowd has previously been studied. In a demonstration, for example, manual head counts at fixed locations were found to be labor-intensive, error prone and cannot account for people leaving a march in front of or entering a march behind a counting location [7]. Even if there are good photographs of a mobile crowd available for a grid/density estimation, the area occupied by a dynamic crowd is difficult to define [6]. All of the above-mentioned methodologies have the additional drawback that they only generate a snapshot view of the crowd size, ignoring its dynamic nature.

As it appears, said methodologies have significant limitations in terms of counting crowds, and are ill-suited to map crowds onto space and/or time due to their single snapshot view. Recent technologies able to register individual movements have the capacity to fill this methodological gap. Several of these tracking technologies in different developmental stages have been used to date. Although computer vision techniques have already been able to reconstruct individual trajectories inside a crowd [15,16], correctly applying these techniques in real-life and large-scale scenarios is still beyond the current state of the art due to several reasons including the necessity of a multitude of camera views, occlusions, variable weather conditions, *etc.* [14]. Other methodologies take advantage of the growing adoption of positioning technologies on modern smartphones, with the most prominent example being the global positioning system (GPS). While this technology is able to deliver fine-grained movement data of individuals, the need for active cooperation of the traced individual—either by installing an application on the smartphone or by distributing logging devices [17]—makes it labor-intensive and less feasible when a representative sample of a large crowd is to be studied. The movement of a mobile device can also be reconstructed by using log files of mobile operators containing information about which cell-towers the device connected to during its lifetime or when calls were made [18,19]. The locational precision of this last methodology (in the order of at least 100 m even in urban settings) is, however, too large for handling the smaller-scale dynamics of crowds.

More recently, Bluetooth has been suggested as an interesting alternative tracking technology. Since the Bluetooth protocol allows for wireless discovery and identification of nearby devices, static Bluetooth sensors placed at strategic locations can give insights into human mobility in a variety of contexts: dynamics at mass events [8,20], urban design [21], social studies [22], travel time estimation of motorized traffic [23], *etc.* Initially envisioned as a low-power and open protocol for implementing Wireless Personal Area Networks by Siemens in 1994, Bluetooth has since become an almost ubiquitous technology on modern mobile devices. Prior to the ability for two devices to connect wirelessly through Bluetooth, one device needs to be discovered by the other. This part of the Bluetooth protocol is called the inquiry phase [24]. The master device transmits inquiry packets, to which discoverable devices within its vicinity respond with inquiry response packets. These include the MAC address (which is a 48-bit identifier of the mobile device), and the class of device (COD) code (which gives a general idea about the type of device and some of its functionalities). By mapping detected MAC addresses to a specific timestamp and location where a sensor that made the discovery was located, one can reconstruct proximity-based trajectories [25]. Since an actual connection is not required, tracked individuals are not aware of the presence of Bluetooth sensors and the methodology is in essence completely unobtrusive. Since Bluetooth 1.2, it is also possible to register the received signal strength indicator (RSSI) of the inquiry response packets, which is loosely correlated with the distance between the sensor and the detected device [26].

In this paper, we propose a novel use-case for the Bluetooth tracking methodology: the mapping of spectators along the track of a road cycling race. We build on the concept of *mobile mapping*, where a combination of a moving platform, navigation sensors and mapping sensors is used for the geo-referenced mapping of information across a study area [27]. Instead of the more traditional imaging sensors, however, we use Bluetooth sensors to detect the proximity of people carrying Bluetooth-enabled mobile phones. The methodology bears resemblance to the popular act of *war driving* where the locations of Wi-Fi Access Points are mapped by a car driving around a study area [28]. The idea of using mobile Bluetooth sensors is not novel as such. Particularly social studies have already embraced the concept of mobile phones as wearable (Bluetooth) sensors for discovering complex social systems [29] or *familiar strangers* as individuals we repeatedly observe yet do not directly interact with [30]. Other studies have already hinted at the possible use for discovering pedestrian travel behavior as well [31]. To our knowledge, however, the concept of using a mobile (Bluetooth) sensor to map a crowd along a trajectory followed by a mobile attractor is without a precedent in scientific literature.

## 3. Methodology and Deployment

**Figure 1.**
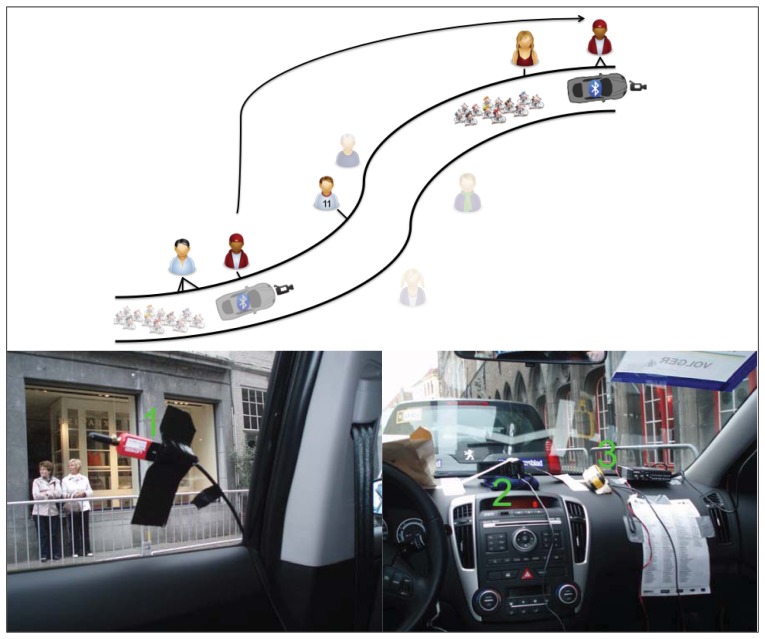
Mobile sensor deployment during the race. Top: schematic overview of the methodology, bottom: actual deployment (1: Bluetooth sensor, 2: video camera, 3: GPS unit).

In order to map spectators, we used a mobile platform equipped with two Bluetooth sensors that moved along the track registering Bluetooth devices belonging to spectators as it passed them by. Figure 1 shows a conceptual representation of the methodology at the top and the used equipment at the bottom. The numbers in the figure correspond to the numbers between brackets in this paragraph. The mobile platform carrying the equipment was a car (Kia Sportage) that belonged to the convoy preceding the racers (on average the racers lagged the platform for between 3 and 6 min). The Bluetooth sensors (SENA Parani UD-100) were attached to the side windows in the back of the car (1). These class 1 Bluetooth devices (*i.e.*, the most powerful class with a theoretical communication range of 100 m) were fitted with an external *stub* antenna with a gain of 1 dBi. Previous experiments had shown that this combination of sensor and antenna is capable of discovering mobile phones at distances of 100 m in a static context. The Bluetooth sensors performed new inquiry scans every 10.24 s. Both sensors were connected to a portable computer (Dell Vostro 3500, Debian Testing OS, kernel 2.6.38-2) running Bluetooth scanning software called *Gyrid* (see https://github.com/Roel/Gyrid, version 0.4.5). This self-implemented framework, built around the *PyBlueZ* (0.18-1) and *BlueZ* (version 4.89-1) frameworks, was also used in previous studies of our research group. Additionally, a video camera (Panasonic HDC-SD10) was installed looking through the front window (2). This way, we could later compare Bluetooth counts with visual counts. Because the recorded video in raw format was too large to fit onto one memory card, two memory cards were regularly swapped and their content copied. Finally, a GPS unit (Garmin GPS60) was used to continuously record the position of the mobile platform (3). A logger on the portable computer registered these locations on the fly. Every time a Bluetooth device was detected, its MAC address, COD code, the RSSI of the detection and the timestamp of the detection were registered. Because of the GPS recordings, every detection at a certain timestamp could later be mapped onto a location along the track followed by the car. Because both the GPS unit as well as the Bluetooth sensors were connected to the same computer, their outputs were automatically synchronized in time. The computer itself was connected to the Internet through a 3G connection and was synchronized with the Network Time Protocol. Synchronization between video data and Bluetooth data was done by manual landmark discovery in the camera footage and linking this to their known position and the time they were passed according to the GPS data. As is indicated in Figure 1 by some of the spectators being mapped onto several nearby positions along the track, some devices are detected more than once during a passing event. Terminologically speaking, these *devices* are associated with several *detections*. This important distinction leads to the concept of a set of (unique) devices and an associated larger set of (non-unique) detections. Additionally, it is known that a minority of spectators will watch the group of cyclists at more than one location along the track. This is depicted by the arrow in the figure. Finally, only a subset of the spectators owns a mobile device with a visible Bluetooth interface and the rest of the crowd is hence not detected (indicated by the transparent icons).

## 4. Case Study: Tour of Flanders 2011

The tour of Flanders (in Dutch: *Ronde van Vlaanderen*) is a one-day road cycling race held yearly in Flanders, Belgium. Due to the popularity of cycling in Belgium and the long history and tradition of the race (the first edition dates back to 1913), the event has become the largest sporting event in Flanders and, as such, has become more or less part of the local cultural heritage. Due to the open nature of the event (the racetrack only traverses public space) little is known about the spatiotemporal characteristics of spectators. Correspondingly, the race was chosen as a fitting case study for the application of Bluetooth tracking as a mobile mapping concept.

**Figure 2.**
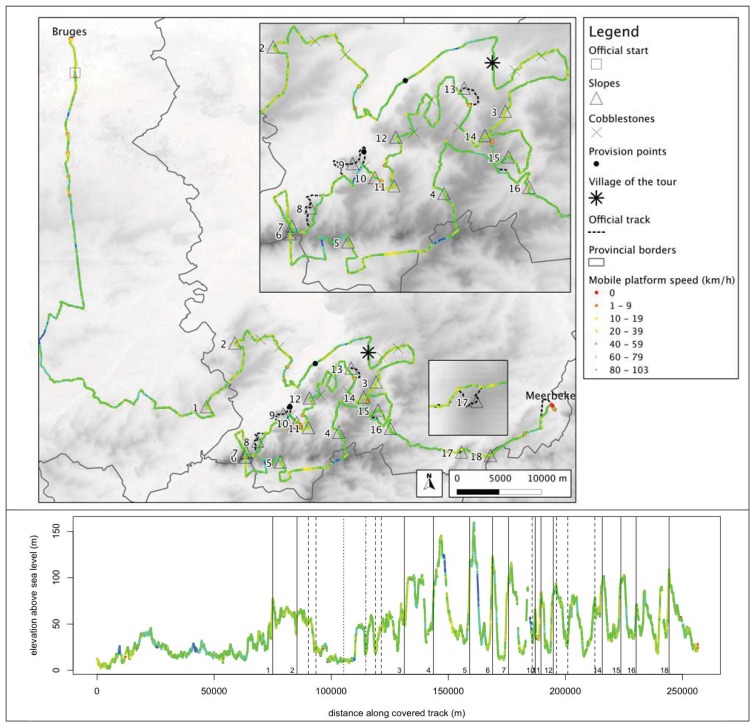
Tour of Flanders, 2011 edition. Top: spatial view of the official track and the trajectory of the mobile platform. Bottom: elevation profile of the mobile platform trajectory (NASA Shuttle Radar Topography Mission elevation data). In both cases, the speed of the mobile platform is shown in a color scale ranging from red (temporary stops) to blue (more than 80 km/h). The 18 official climbs are shown as triangles in the spatial view and solid vertical lines in the elevation profile (non covered climbs excluded). The dashed vertical lines represent the cobblestoned segments, the dotted vertical line the first provision point, and the dashed-dotted line the village of the tour.

An overview of the trajectory followed by the mobile platform—including its speed—is given in Figure 2. The 2011 edition started in Bruges at 9:45 AM and ended in Meerbeke (winner: 16:00 AM, last rider: 16:18 AM), taking the cyclists over 256.3 km and 18 slopes. For safety reasons, some sections of the race could not be accessed by four-wheeled vehicles. Therefore, the trajectory of the mobile platform carrying the Bluetooth sensors in some places deviates from the official track. This was the case around four slopes (8, 9, 13 and 17). As a consequence, the crowds gathered at these locations were not covered by the platform. There were two feed zone points along the track where racers were provided food and drinks.

## 5. Results

### 5.1. Prior Experiments with Mobile Platform

**Figure 3.**
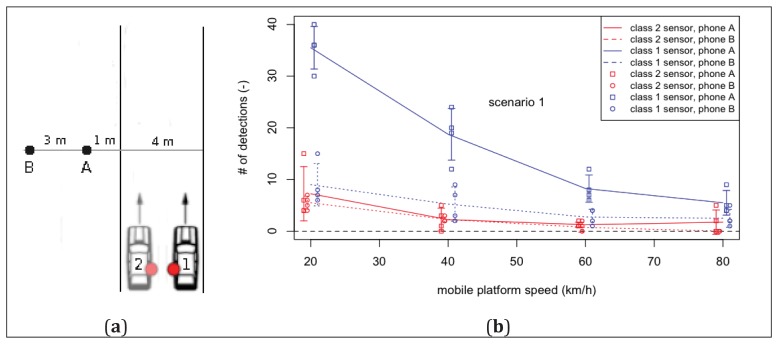

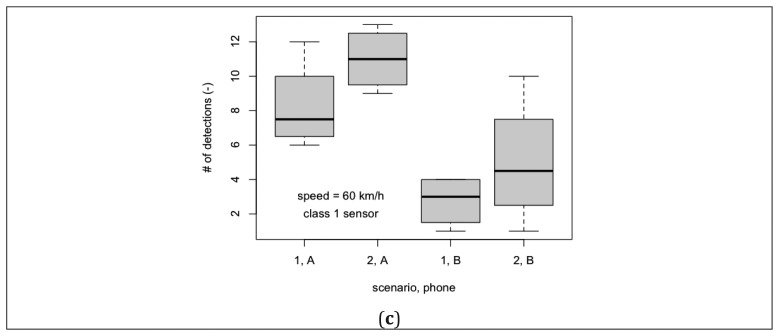
Mobile Bluetooth detection process investigation: (**a**) experimental setup showing both mobile phones (A, B) and the two passing scenarios (1: sensor facing phones, 2: sensor not facing phones); (**b**) effect of sensor type, speed, and phone distance (both the mean and standard deviations, as well as the individual values of all combinations are shown; both error bars and individual data points are offset from their real x-value for visual clarity); (**c**) effect of sensor placement on mobile platform at a speed of 60 km/h.

Prior to deploying the mobile platform, the Bluetooth detection process and its sensitivity towards several factors in a mobile context needed to be investigated. Five factors were studied: (1) the type of Bluetooth sensor, (2) its position on the mobile platform, (3) the distance between the detectable mobile phone and the road covered by the mobile platform, and (4) the speed of the mobile platform. A small-scale experiment was conducted under controlled conditions by placing two discoverable Bluetooth-enabled phones (representing two spectators) next to a road section at different distances (resp. 1 and 3 m perpendicular distance, at 1 m above ground), controlling the different factors and calculating the number of detections of each phone during a passing event of the mobile platform. For every combination of factors, four passage runs were made. The main and concise goal of this experiment was to check the feasibility of the proposed methodology before deploying it on a larger scale. A view of the experimental setup is given in Figure 3(a). The influence of the sensor type, the mobile platform speed and the distance between the road and the mobile phone can be seen in Figure 3(b). The difference in performance between the class 2 and class 1 sensor is evident. Where the class 2 sensor already missed the closest phone once at 40 km/h (the furthest phone was even missed on every run at 80 km/h), the class 1 sensor did not miss any phone on any run. The mobile platform speed influences the number of detections in a negative way. More importantly, though, it does not seem to form a bottleneck when using a class 1 sensor (at least for speeds not surpassing 80 km/h). The difference between both phones is manifest in the case of the class 1 sensor: a larger distance lowers the number of detections.

With a class 2 sensor, the difference is nearly negligible. Again there does not seem to be an indication, however, that this distance blocks the methodologies usability. Next, the position of the sensor on the mobile platform needed to be investigated. Since it was not possible to mount an antenna on the rooftop of the car, it had to be placed inside of the car. Prior experiments had shown the rear side window being the best location in contrast to the ceiling or the front windshield (not shown). It was not known, however, what the effect was of the sensor either facing or not facing the side of the road with detectable phones. This was tested by performing passage runs at a speed of 60 km/h under both scenarios (Figure 3(c)). Contrary to what could be expected, both phones were detected more often when the sensor was not facing them (scenario 2) although the difference is clearly not statistically significant. The difference between both phones is more pronounced, showing that the effect of the distance is larger than that of the sensor placement in the car. In order to minimize the probability of missing detectable phones, the mobile platform was equipped with a class 1 sensor on each side during the actual case study experiment described in the next section.

### 5.2. Case Study

#### 5.2.1. Pre-Processing and Mapping of Detections along Trajectory

During the course of the race, two types of log files were generated. First, each Bluetooth sensor generated a Bluetooth log file containing one log line per detection in the following format: *timestamp,MAC-address,COD-code,RSSI* (e.g., *20110403-101520,00:12:34:56:78:9A,5898756,-72*). Second, the sequential GPS fixes were gathered on the portable computer and written to a separate log file. In order to geo-localize the Bluetooth detections, both data sources needed to be combined. First of all, the Bluetooth detections picked up before the GPS unit started recording were deleted. Subsequently, the data were imported in the R suite (http://www.r-project.org, version 2.14.0) and transformed to two data frames (one for the Bluetooth data of both sensors merged together and another for the GPS data). Table 2 lists some of the main characteristics of both data frames. Given that the GPS unit had a sampling rate of 1 second and the Bluetooth detections had a temporal precision of 1 second as well, both data frames could be merged into one data frame based on their respective timestamps. As such, each Bluetooth detection was given a location (both an xy-location and a distance along the trajectory followed by the mobile platform). Finally, we examined the device classes detected by the sensors and found that roughly three out of four detections were from phones, and one out of four were audio/video devices. Other types (including devices which did not give COD information) were very sparse. Only phones can be more or less directly linked to a physical person. So in order to map only spectators, we filtered out the 15,597 phones (which amounted to 130,464 detections in the dataset). The set of audio/video devices consisted almost entirely of handsfree devices such as car kits and represent vehicles rather than persons. They were excluded from the rest of the analyses together with the other less frequent device classes.

#### 5.2.2. Crowdedness along Trajectory

**Figure 4.**
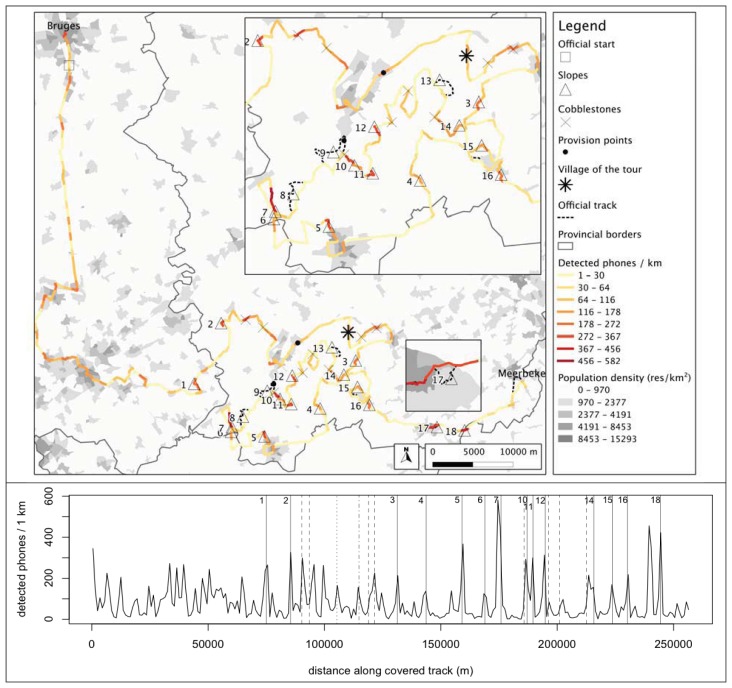
Number of detected phones along 1 km long segments of the trajectory followed by the mobile platform as an indicator of crowdedness. Top: spatial view using a yellow-to-red color scale to depict the number of phones, class breaks according to the Jenks natural breaks optimization [32]. The background shows the population density of statistical sectors in the area, colored in a grey-scale, once again according to Jenks natural breaks. Bottom: one-dimensional view.

In order to map spectators along the trajectory, we divided the trajectory into 1 km long segments. For each segment, we aggregated the Bluetooth detections that occurred in the same time span and calculated the number of unique MAC addresses. This way we could map the number of detectable spectators along the course of the trajectory as an indicator of local crowdedness. The result is shown in Figure 4. The two-dimensional spatial view and one-dimensional cross section show alternating zones of higher and lower numbers of spectators. Most of the densely crowded segments coincide with the slopes along the track. Slope number 7 (*Oude Kwaremont*) clearly attracted the largest number of detectable spectators (582 phones over 1 km). The other crowded segments are either associated with cobblestoned segments, or villages or cities (visible as concentrations with high population densities) that are located on the track.

#### 5.2.3. From Bluetooth Devices to Crowd Size

In the previous section, we have identified crowded zones according to the number of phones detected over a certain distance. As stated in the introduction, however, we intend to make reasonable estimations of the size of an entire crowd. As a consequence, we have to transform the number of detected devices into an approximate number of spectators. In order to do this, we need to know how large the share of detectable persons in a crowd is. We call this the *detection ratio*, and calculate it by counting the number of (unique) detected devices and visual head counts during a certain time period, and dividing the former by the latter. In case of static Bluetooth sensors, a person usually sits in the close vicinity of a sensor and counts the number of people passing by. Using this methodology, a previous study with a dataset gathered in 2010 delivered a detection ratio of 11.0 ± 1.8% [8]. Visually counting people on the side of a road from a mobile platform is less trivial than counting moving people from a static position. Nevertheless, the video footage recorded from the mobile platform could be used to count spectators visible from the platform as it passed by. In total, 14 calculations were made along the trajectory covering 52.2 km in total (20% of the total trajectory length). These are shown in Table 3. If all measurements are taken into account, the average detection ratio lies at 14.3 ± 3.9%. The resulting relative standard error RSE, which represents the relative error the crowd size estimation will have, is 27.3%. If the two measurements with abnormally high detection ratios (measurements 6 and 9 both lie above *Q3* (the third quartile) + *IQR* (interquartile range)) are regarded as outliers and excluded, we end up with an average detection ratio of 13.0 ± 2.3% (RSE of 17.9%). Further clarification as to why these two values can be considered outliers is given in the discussion. The table also includes the detection ratio of a previous experiment with static Bluetooth sensors ([8], RSE of 16.4%). Using these detection ratios, the extrapolated total crowd size at the segment with the largest number of detected phones along the trajectory lies at 4,070 ([3,198–5,596], with outliers) or 4,477 ([3,804–5,439], without outliers). We can use these figures to roughly get an idea about the spectator density along the road. Taking into account the length of this segment (1,000 m) and the fact that spectators can line up on both sides of the road, we end up with roughly 2 spectators per meter of roadside. Analogously, the estimate of the total crowd size that was covered by the mobile platform during the entire duration of the measurements lies at 109,070 ([85,720–149,957], with outliers) or 119,977 ([101,549–145,937], without outliers). This represents roughly one spectator every 2 m.

#### 5.2.4. Mobile Platform Speed Influence under Real-Life Conditions

**Figure 5.**
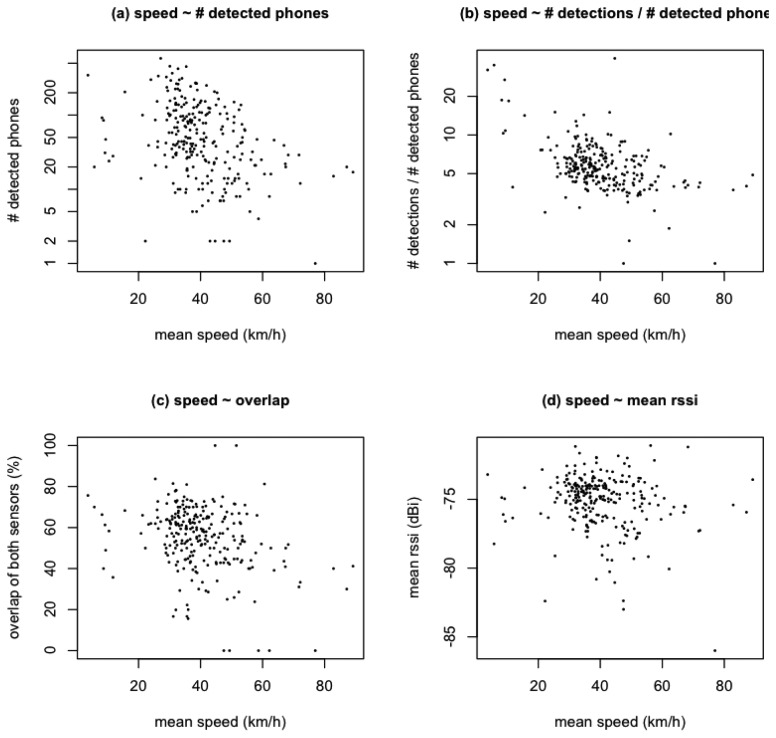
Real-life influence of mobile platform speed on four trajectory segment characteristics: (**a**) number of detected phones (*log scale*), (**b**) number of detections/number of detected phones (*log scale*), (**c**) sensor overlap, (**d**) mean RSSI.

Since the Bluetooth sensors were placed on a mobile platform, we need to investigate whether the speed with which the platform moves along the trajectory has any influence on the spectator detection process. Preliminary experiments prior to the actual case study already demonstrated that the influence of the speed is negligible under idealized circumstances (Section 5.1). The dataset from the cycling race should, however, be explored as well in order to confirm or deny this finding. Four characteristics were calculated for each 1 km segment of the trajectory (as depicted in Figure 4), and correlated with the mean speed attained over each segment. The resulting scatter plots are shown in Figure 5.

First, the number of detected phones over a segment is compared to the mean speed of the platform over that segment (Figure 5(a)). The goal of this visualization is not to test for a direct link between both values (this assumption is not realistic anyway), but to focus on the segments at the low and high extremes of the speed spectrum. The segments with mean speeds over 80 km/h are still linked to between 10 and 20 detected phones, while the slowest segments are not associated with extremely high numbers of detected phones. Consequently, we did not find an indication that very low or very high speeds bias the detection process in a significant way. Subsequently, the ratio between the number of detections and the number of detected phones is compared to the mean speed of each segment (Figure 5(b)). Generally speaking, a higher ratio corresponds to phones being detected more times during a passing event (a higher sampling frequency) leading to a smaller likelihood of it being missed by the Bluetooth sensor passing it by. The very slow segments seem to correspond to slightly higher ratios, but the very fast segments have ratios that lie within the range of ratios for average speeds. This constitutes an extra indication that high speeds do not hinder the detection process in a significant way. The overlap between both sensors on the mobile platform (defined as the ratio of the size of the intersection of the sets of detected phones by each sensor to the size of the union of both device sets) seems to roughly lie between 20% and 80% (Figure 5(c)). Neither the slowest nor the fastest segments exhibit overlaps that deviate consistently with the general average. Finally, the mean of all RSSI values gathered in each segment is also plotted against the mean speed (Figure 5(d)). Again, there is no indication of the signal strength of detections being influenced by the speed of the platform carrying the sensors.

## 6. Discussion

A crowd of spectators watching a road cycling race was mapped by a mobile platform equipped with two Bluetooth sensors. The locations of nearly 16,000 detected phones carried by the spectators were used as an indicator of the crowdedness levels along the race trajectory. Figure 4 shows that the methodology was able to distinguish crowded zones from zones with sparse numbers of spectators. Nearly all hotspots correspond to either slopes, cobblestoned segments or—in a lesser degree—urbanized areas. After careful examination of this map, the research team present during the race and the race organizers agreed that the results were consistent with their own experience.

Clearly, this reliability analysis cannot be constricted to personal (and possibly subjective) experience. Consequently, the camera footage from aboard the mobile platform was used as an additional (non-quantitative) ground truth data source. As an additional advantage of this data source, segments of the trajectory can be subjected to visual head counts forming smaller but quantitative data sources. By comparing these visual head counts with the number of detected phones, a detection ratio was calculated along 14 of these segments. This resulted in an average detection ratio of 14.3 ± 3.9% (RSE of 27.3%). Most of the segments exhibited a detection ratio slightly higher than the reference detection ratio from a previous study performed in a static context (±11%), while two segments showed unusually high detection ratios above 20%. There are two possible explanations for this. First, it is possible that some phones located along neighboring segments are included in the detection ratio calculation while the visual head counts are restricted more strictly to the 1 km segment. This may lead to a higher enumerator and thus a higher detection ratio. Since we anticipated this effect when selecting the segments for visual head counts, the selected segments did not exhibit large crowds at their borders and therefore the effect should not be significant. A second and probably more significant effect is due to *invisible* spectators. Spectators who are missed for the visual head counts (e.g., in buildings) can also lead to an overestimation of detection ratios. This effect is much harder to control with an appropriate choice of segments because visual confirmation with the camera footage is impossible. The very high detection ratios are almost certainly caused by this effect. Correspondingly, an average detection ratio of 13.0 ± 2.3% (RSE of 17.9%) is attained after pruning of the two most extreme outliers.

As already pointed out in the previous paragraph, we should consider the fact that a 1,000 m long segment may actually cover a larger detection area. In the worst case this could measure up to 1,200 m if the Bluetooth sensors would have a 100 m detection range in a mobile context as well. The overlap between each pair of consecutive segments (defined as the ratio of their intersection and union) was extracted from the dataset. More than 90% of the pairs have overlaps smaller than the worst-case overlap of 10% ((100 m + 100 m)/(1,000 m + 1,000 m)). While the overlap between segments should not be completely disregarded, the effect will be of minor importance on larger scales.

**Figure 6.**
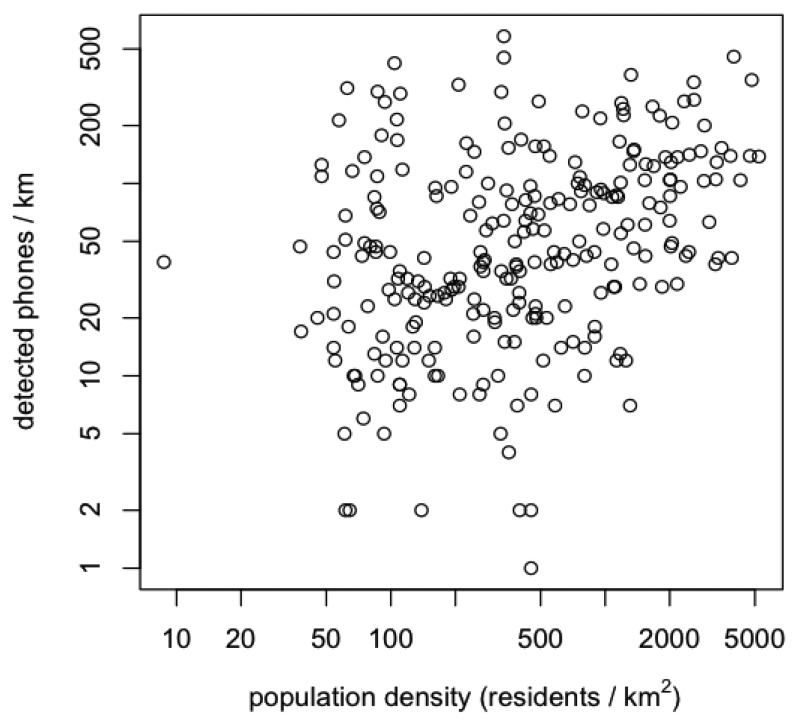
Scatter plot of statistical population density *versus* number of detected phones over each 1 km long segment of the trajectory.

An additional concern affecting the reliability is the selectivity of the detection process towards spectators of the race instead of the more general public (the entire race takes place on the public domain). In the worst case, the mobile platform could be mapping the population density because of a disproportionally large detection range. As a first way of evaluating to what extent this is the case, we included the population density as a background layer in Figure 4. While the generally higher numbers of detected devices in the south-western part of the map view could be linked to the locally higher population densities, most of the crowded zones further along are situated in locations with very low statistical population densities. Figure 6 shows a scatter plot of population density, calculated for each segment by a spatial join operation in ArcGIS returning the mean if a segment crossed more than one population density area, *versus* the number of detected phones along each 1 km long trajectory segment. There is no clear correlation between both variables, which is another indication that there is a high selectivity towards spectators of the race. In the end, however, the correct counting of a crowd also raises an ontological issue linked to a specific scenario: how do you define a spectator of the race (e.g., solely by his/her proximity to the race track or also by additional constraints?).

It is worthwhile comparing the Bluetooth tracking methodology with other and more often applied methodologies. Relevant literature [6] cites a relative standard error of the order of 10% for the grid/density method, seemingly making our methodology with a RSE of 17.9% less accurate. It should be stated, however, that it is first not always clear which ground-truth literature citations are based on. Additionally, the real merit of the proposed methodology is not just the counting of a crowd as such, but also its ability to easily map its behavior over time and space. As demonstrated in this study, Bluetooth tracking is able to generate insightful information (maps) of a crowd, its (varying) location, and size. With slight modifications, the same methodology could be used for crowds dispersed over more complex study areas. A mobile platform (or more than one) could also re-visit the same locations regularly for temporal pattern detection, or they could be combined with static sensors.

While the results were satisfactory in the context of this case study, other studies might necessitate more accurate counts and/or localizations of detections. Further calibration of the sensors will be necessary to accomplish this goal. The rather low overlap between both sensors on the platform, as seen in Figure 5, seems to indicate that a higher number of sensors/platforms might also play a beneficial role. Additionally, smaller scale investigations (in contrast with the larger-scale approach adopted in this case study) will need more certainty on the locational accuracy of the detections along the road section. The potential overlap between the detection areas of subsequent segments should also be further examined.

## 7. Conclusions/Outlook

In this paper, we demonstrated the added value of Bluetooth technology in the mobile mapping of individuals. The spectators of the Tour of Flanders 2011 were mapped along the trajectory followed by a mobile platform equipped with Bluetooth sensors. Nearly 16,000 devices were detected along 256 kilometers. Dividing the trajectory into 1 km segments, we were able to identify crowded hotspots in a detailed manner. These were mostly situated along slopes and cobblestoned segments, as anticipated by both the research group and the race organizers. Comparing the Bluetooth detection data with video recordings from the platform, an average detection ratio of 13.0 ± 2.3% was calculated. The relative standard error of 17.9% is slightly higher than that of some alternative methodologies, but still acceptable for most purposes.

Our results showed that the main benefit of the proposed methodology is its ability to generate highly detailed spatiotemporal information on crowds with relative ease and without the need for expensive equipment or cooperation of the crowd. Despite these promising first results, some aspects were identified that need further investigation. A rather low overlap between the devices detected by both sensors on the mobile platform points out that a better calibration is necessary to further enhance the reliability of the detection process and its locational accuracy.

## Figures and Tables

**Table 1.** Characterization of crowd scenarios according to the mobility of the attendees and the presence/mobility of an attractor. The attractors for the specific examples are shown between brackets.

		**Attractor**		
		Static	Dynamic	No attractor present
**Attendees**	Static	inauguration (president), football game (pitch)	cycling race (cyclists), papal visit (pope-mobile)	New Years' celebration
	Mobile	riot (police forces)	love parade (trucks with music installations)	marathon, demonstration

**Table 2.** Data pre-processing summary.

**Bluetooth**	First detection	09:34:06	
	Last detection	16:13:57	
	Number of detected devices	16,182	
	Number of detections	177,079	

**GPS**	First fix	09:34:06	
	Last fix	16:14:46	
	Total distance	256,668 m	

**Video**	Start	09:40:51	
	End	15:52:50	
	Blind spots (swapping of memory cards)	± 30 min	

**Filtering**	**Device class**	**# of detections**	**# of detected devices**

	Phone	130,464 (73.7%)	15,597 (96.4%)
	Audio/Video	45,073 (25.5%)	302 (1.9%)
	Unknown	787 (0.4%)	124 (0.8%)
	Computer	598 (0.3%)	136 (0.8%)
	Network access point	122 (0.1%)	16 (0.1%)
	Imaging	35 (0.0%)	7 (0.0%)

**Table 3.** Detection ratios along the trajectory, calculated by comparing the numbers of detected Bluetooth phones with visual spectator counts from video recordings. The two greyed out rows represent abnormally high detection ratios and can be regarded as outliers. The bottom part of the table shows the average detection ratios, standard deviations and relative standard errors with and without pruning of the outliers.

**Number**	**Duration (s)**	**Distance (m)**	**Visual count (-)**	**Detected phones (-)**	**Detection ratio (%)**	**Average (%)**	**St. dev. (%)**	**Rel. st. error RSE**	
1	447	3,730	1,984	253	12.75%	14.3	3.9	27.3	average detection ratio based on all measurements
2	527	4,246	1,350	153	11.33%	**13.0**	**2.3**	**17.9**	**average detection ratio with measurements 6 and 9 pruned**
3	1,035	11,694	4,567	580	12.70%	*11.0*	*1.8*	*16.4*	*reference detection ratio from literature [8]*
4	286	4,909	1,919	313	16.31%				
5	144	3,148	1,013	116	11.45%				
**6**	**143**	**1,729**	**1,034**	**220**	**21.28%**				
7	358	4,479	1,502	154	10.25%				
8	439	5,925	4,183	515	12.31%				
**9**	**275**	**2,777**	**1,207**	**273**	**22.62%**				
10	261	2,547	1,320	175	13.26%				
11	143	1,755	1,758	209	11.89%				
12	156	1,691	970	141	14.54%				
13	152	2,442	808	90	11.14%				
14	123	1,155	496	91	18.35%				
